# Rethinking Antibiotic Prophylaxis in Femoral-Access Catheter Ablation: A Pragmatic, Risk-Based Proposal

**DOI:** 10.3390/jcm15051783

**Published:** 2026-02-27

**Authors:** Amedeo Prezioso, Domenico Pecora, Andrea Dell’Aquila, Cristina Seguiti, Paolo Colombini, Carmelo La Greca

**Affiliations:** 1 Cardiac Electrophysiology Unit, Cardiology Department, Fondazione Poliambulanza Istituto Ospedaliero, 25124 Brescia, Italy; domenico.pecora@poliambulanza.it (D.P.); andrea.dellaquila@hotmail.com (A.D.); carm.lagreca@gmail.com (C.L.G.); 2 Infectious Diseases Unit, Fondazione Poliambulanza Istituto Ospedaliero, 25124 Brescia, Italy; cristina.seguiti@poliambulanza.it (C.S.); paolo.colombini@poliambulanza.it (P.C.); 3 General Medicine and Geriatrics Unit, Fondazione Poliambulanza Istituto Ospedaliero, 25124 Brescia, Italy

**Keywords:** catheter ablation, antibiotic prophylaxis, infectious complications, femoral venous access, antimicrobial stewardship

## Abstract

Catheter ablation is widely performed for the treatment of supraventricular and ventricular arrhythmias; however, specific recommendations regarding antibiotic prophylaxis for femoral-access procedures are lacking in current international guidelines, resulting in substantial practice variability. Although infectious complications are rare, severe events such as infective endocarditis and septic complications have been reported and are associated with significant morbidity and mortality. At the same time, increasing concerns regarding antimicrobial resistance challenge the indiscriminate use of prophylactic antibiotics in low-risk settings, underscoring the need for a more selective and rational approach. This narrative review summarizes the available evidence on infectious complications related to femoral-access catheter ablation and electrophysiological studies. In the absence of prospective data, indirect evidence and biological plausibility were integrated to inform clinical decision-making. Based on multidisciplinary collaboration, we propose a pragmatic, risk-based institutional protocol that discourages routine prophylaxis in low-risk patients while allowing selective antibiotic use in predefined high-risk scenarios. This framework aims to balance patient safety with antimicrobial stewardship and may help reduce unwarranted variability in contemporary electrophysiology practice.

## 1. Introduction

Catheter ablation has become a cornerstone in the management of both supraventricular and ventricular arrhythmias, with a continuously increasing number of procedures performed worldwide. Most electrophysiological studies and catheter ablations are routinely carried out through femoral venous access, often requiring multiple vascular sheaths and prolonged intracardiac manipulation [1].

Although the overall rate of infectious complications following catheter ablation is extremely low, severe infectious events, including infective endocarditis and septic embolic complications, have been reported and are associated with substantial morbidity and mortality, while large observational studies confirm a very low incidence of such events in contemporary practice [2,3,4,5].

Current international guidelines and consensus documents on catheter ablation provide limited or no specific recommendations regarding antibiotic prophylaxis in this setting. As a result, clinical practice varies widely among institutions, ranging from systematic antibiotic administration to complete omission of prophylaxis [6,7].

Similarly, regional perioperative antibiotic prophylaxis guidelines do not specifically address transcatheter catheter ablation or electrophysiological procedures, further highlighting the lack of standardized recommendations in this field [8].

In the absence of randomized controlled trials and robust evidence, a pragmatic and risk-based approach may represent a reasonable strategy to balance patient safety with principles of antibiotic stewardship. This narrative review aims to summarize the available evidence on infectious complications related to femoral-access catheter ablation and electrophysiological studies, and to propose a rational, risk-based protocol for antibiotic prophylaxis.

## 2. Search Strategy and Study Selection

A focused narrative review of the literature was conducted to identify publications addressing infectious complications and antibiotic prophylaxis in the setting of catheter ablation and electrophysiological studies performed via femoral venous access. For the purpose of this manuscript, a narrative review is defined as a structured, non-systematic synthesis of heterogeneous evidence aimed at contextualizing clinical reasoning and identifying practical implications, rather than providing quantitative pooled estimates. The search and study selection were independently performed by three investigators, including two electrophysiologists and one infectious diseases specialist, with discrepancies resolved by consensus.

Searches were conducted using PubMed and Embase, with the aim of identifying international guidelines and expert consensus documents, as well as case reports and case series describing infective endocarditis, bacteremia, sepsis, or other severe infectious complications temporally associated with catheter ablation procedures.

Studies focusing exclusively on surgical ablation or cardiac implantable electronic device implantation were excluded. Reference lists of relevant articles were manually reviewed to identify additional pertinent reports.

## 3. Current Guidelines and Consensus Documents

International guidelines and expert consensus documents represent the main reference for procedural standards in catheter ablation; however, they provide limited guidance regarding antibiotic prophylaxis in this setting.

The 2024 ESC Guidelines for the management of atrial fibrillation comprehensively address patient selection, procedural techniques, and peri-procedural management, but do not include specific recommendations on antibiotic prophylaxis for catheter ablation procedures [1]. Similarly, the 2024 EHRA consensus document on catheter and surgical ablation of atrial fibrillation focuses on procedural safety and complication management, without addressing the role of routine or selective antibiotic prophylaxis [7].

A comparable lack of standardized recommendations is observed in documents dedicated to ventricular arrhythmia ablation. In the 2019 HRS/EHRA/APHRS/LAHRS expert consensus statement on catheter ablation of ventricular arrhythmias, prophylactic antibiotics are not generally recommended for sterile procedures such as ventricular arrhythmia ablation [6]. However, the document acknowledges substantial heterogeneity in clinical practice: approximately 40% of the writing committee members administer prophylactic antibiotics to patients with pacemakers or implantable cardioverter-defibrillators undergoing ventricular arrhythmia ablation, while about 25% never do, and the remainder selectively use prophylaxis based on perceived risk factors for device infection. In addition, around 30% of the writing committee members report using antibiotic prophylaxis in the setting of epicardial access. Importantly, no specific antibiotic regimen is recommended, reflecting the absence of supporting evidence.

At a regional level, perioperative antibiotic prophylaxis guidelines are primarily designed for surgical and selected interventional procedures. Notably, regional guidelines on perioperative antibiotic prophylaxis do not specifically address transcatheter catheter ablation or electrophysiological studies [8].

## 4. Reported Infectious Complications Following Catheter Ablation

Infectious complications following catheter ablation are exceedingly rare; however, when they occur, they may lead to severe and potentially life-threatening clinical outcomes. Available evidence on this topic is largely limited to isolated case reports and small case series, reflecting both the low incidence of these events and the absence of systematic prospective data.

Stec et al. reported a case of infective endocarditis occurring after ventricular arrhythmia ablation in a patient with an implantable cardioverter-defibrillator, complicated by a complex clinical course and requiring prolonged antimicrobial therapy [3]. Importantly, the authors explicitly stated that no peri-procedural antibiotic prophylaxis was administered. This case highlights the potential vulnerability of patients with intracardiac material to bacteremia following extensive intracardiac manipulation.

Similarly, Udongwo et al. described a case of iatrogenic infective endocarditis with septic emboli following intracardiac manipulation during catheter ablation [2]. Despite the absence of procedural complications, the patient developed a severe systemic infection, and peri-procedural antibiotic prophylaxis was not reported.

Infective endocarditis has also been described after atrial fibrillation ablation in patients without overt immunosuppression. Weis et al. reported a case of septic vegetation at the left atrial appendage entrance following pulmonary vein isolation, again in the absence of reported peri-procedural antibiotic prophylaxis [9].

In the reported cases, the causative pathogens were predominantly Gram-positive organisms, particularly *Staphylococcus aureus* and the viridans group streptococci, consistent with transient bacteremia originating from skin and inguinal microbial flora during vascular access and intracardiac manipulation [2,3,9]. The femoral region is characterized by higher bacterial colonization compared with other vascular access sites, including both Gram-positive cocci and selected Gram-negative organisms. Although less frequently described in the available reports, Gram-negative pathogens have also been implicated in selected cases, further supporting the biological plausibility of bloodstream contamination originating from the inguinal access site.

Beyond isolated reports, limited data from larger cohorts further confirm the rarity of infectious complications. In a meta-analysis by Benali et al., infectious events following atrial fibrillation ablation were classified among severe procedural complications but occurred with a cumulative incidence of <0.06% within this category. Importantly, these events were not significantly associated with ablation strategy, energy source, or the use of peri-procedural antibiotic prophylaxis [4].

Consistently, in a large real-world analysis of 14,875 inpatient atrial fibrillation ablation procedures from the U.S. National Inpatient Sample, Wu et al. reported systemic infectious complications (sepsis or bacteremia) in approximately 0.3% of cases, without identifiable procedure-specific predictors uniquely associated with infection [5].

Although mechanistically distinct, other severe septic complications following catheter ablation have been reported. Atrio-esophageal fistula after atrial fibrillation ablation has been associated with mediastinitis, sepsis, and high mortality [10].

Overall, available evidence indicates that infectious complications after catheter ablation occur infrequently but may be associated with substantial morbidity and mortality. Notably, in reported cases and available cohort data, peri-procedural antibiotic prophylaxis was either not administered, not reported, or not shown to be associated with a reduced incidence of infectious events.

## 5. Femoral Venous Access and Risk of Bacteremia

Although direct evidence linking femoral venous access to infectious complications specifically in catheter ablation procedures is lacking, indirect data from the vascular access literature suggest a higher risk of catheter-related bloodstream infections associated with femoral access compared with other venous sites [11].

In a prospective observational study conducted in the intensive care setting, Lorente et al. reported a higher incidence of catheter-related bacteremia with femoral compared with internal jugular venous access, with rates of 9.52 versus 4.83 per 1000 catheter-days, respectively [12].

Similarly, in a systematic review and meta-analysis evaluating catheter-related bloodstream infections, O’Horo et al. demonstrated a significantly increased infection risk associated with femoral access, with femoral catheterization conferring an approximately twofold higher risk of bloodstream infection compared with alternative access sites [13].

Although these studies were not conducted in the context of electrophysiological procedures, they provide a biologically plausible framework suggesting that the femoral insertion site represents a clinically relevant risk factor for transient bacteremia. In the setting of catheter ablation procedures, which often involve multiple femoral sheaths and prolonged intracardiac manipulation, this indirect evidence supports consideration of femoral access as a potential contributing factor to bloodstream contamination.

Importantly, these observations do not justify routine antibiotic prophylaxis for all patients undergoing femoral-access catheter ablation. Rather, they support the rationale for a selective, risk-based approach in which femoral access is considered alongside patient-related risk factors when evaluating the potential need for peri-procedural antibiotic prophylaxis.

## 6. A Risk-Based Approach to Antibiotic Prophylaxis

Given the extremely low incidence of infectious complications following catheter ablation and the lack of randomized or prospective evidence supporting routine antibiotic prophylaxis, a universal prophylactic strategy cannot be justified.

Beyond the limited data on efficacy, increasing concerns regarding antimicrobial overuse and the global rise in antibiotic resistance represent a key driver for re-evaluating routine prophylaxis in electrophysiology procedures. The indiscriminate administration of antibiotics in low-risk settings contributes to selective pressure, promotes antimicrobial resistance, and exposes patients to unnecessary adverse effects, conflicting with established principles of antimicrobial stewardship [14,15].

A selective, risk-based approach aims to balance these concerns by identifying clinical scenarios in which the potential consequences of transient bacteremia may be more severe. Patient-related and procedural factors may be taken into account to guide individualized decision-making.

## 7. A Multidisciplinary Institutional Protocol Proposal for Antibiotic Prophylaxis in Catheter Ablation

**Figure 1:**
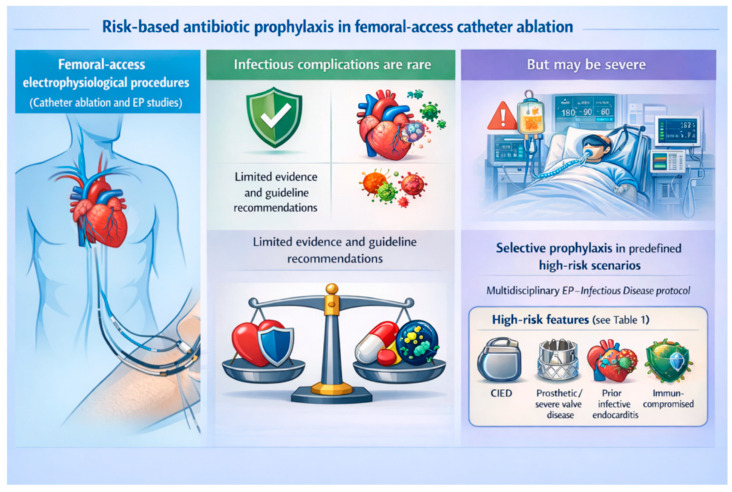
A graphical overview of the proposed risk-based approach to antibiotic prophylaxis in femoral-access catheter ablation.

Based on the available literature and multidisciplinary discussion with infectious diseases specialists, we propose a pragmatic, risk-based institutional protocol for antibiotic prophylaxis in femoral-access catheter ablation and electrophysiological studies. The protocol is designed to discourage routine prophylaxis in low-risk patients, while allowing prophylaxis to be considered in selected high-risk scenarios (Figure 1).

### 7.1. Routine Prophylaxis

Routine antibiotic prophylaxis should not be performed in femoral-access catheter ablation and electrophysiological studies.

### 7.2. Indications for Prophylaxis

Antibiotic prophylaxis may be considered in selected patients deemed at high infectious risk, defined by the presence of at least one of the criteria summarized in Table 1. The high-risk features listed in Table 1 were identified based on biological plausibility, available indirect evidence, and multidisciplinary discussion, and should not be interpreted as absolute indications for antibiotic prophylaxis.

### 7.3. Recommended Antibiotic Regimens

**Figure 2:**
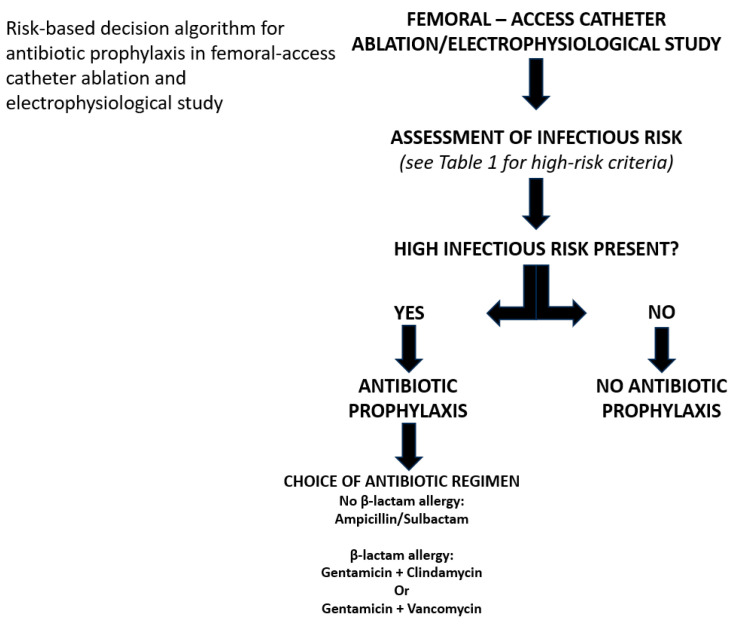
A step-by-step decision algorithm summarizing the proposed approach.

When prophylaxis is considered appropriate, a reasonable first-choice regimen may be ampicillin/sulbactam 2 g + 1 g IV administered 30–60 min before the procedure. In patients with β-lactam allergy, alternative regimens may include clindamycin plus gentamicin or vancomycin plus gentamicin, in accordance with regional perioperative prophylaxis recommendations for transfemoral cardiac interventions and local antimicrobial stewardship policies, taking into account contemporary infective endocarditis prophylaxis guidance [16,17]. The proposed decision algorithm is summarized in Figure 2.

### 7.4. Rationale for Antibiotic Selection

In high-risk patients undergoing femoral-access procedures, ampicillin/sulbactam was selected to provide broader antimicrobial coverage against Gram-positive skin flora and selected Gram-negative organisms relevant to the inguinal region, compared with narrower-spectrum agents. The femoral access site is characterized by higher bacterial colonization, including enteric Gram-negative species, which may not be fully addressed by narrower-spectrum agents alone. First-generation cephalosporins (e.g., cefazolin) are widely used for prophylaxis in clean surgical procedures and CIED implantation across many European centers. However, in the specific context of femoral venous access—where higher bacterial colonization and potential Gram-negative involvement may be relevant—their narrower Gram-negative coverage could represent a limitation in selected high-risk patients. Therefore, antibiotic selection should be tailored to local microbiological epidemiology and institutional protocols [18,19].

This choice is consistent with regional perioperative prophylaxis recommendations and with antimicrobial strategies adopted in other transfemoral structural interventions (e.g., transcatheter aortic valve implantation), where broader coverage is considered appropriate given the access site [8].

## 8. Discussion

Infectious complications following femoral-access catheter ablation and electrophysiological studies are rare; however, when they occur, they may be associated with significant morbidity and mortality. Despite the widespread use of catheter ablation, current international guidelines and expert consensus documents provide limited guidance on antibiotic prophylaxis in this setting, resulting in substantial heterogeneity in clinical practice [1,6,7].

Available evidence on infectious complications after catheter ablation is largely derived from isolated case reports and small case series, with only limited data from large observational cohorts. Although a direct causal relationship cannot be established, these reports often describe severe clinical presentations occurring in patients with predisposing conditions such as intracardiac devices or structural heart disease [2,3,4,5,9]. This observation suggests that selected patient populations may be more vulnerable to the consequences of transient bacteremia associated with extensive intracardiac manipulation.

At the same time, the routine use of antibiotic prophylaxis in all patients undergoing femoral-access catheter ablation cannot be justified in the absence of supporting evidence. Indiscriminate antibiotic administration conflicts with established principles of antimicrobial stewardship and may contribute to antimicrobial resistance, drug-related adverse events, and unnecessary healthcare costs [14,15,20].

In this context, a pragmatic, risk-based approach appears reasonable. Rather than advocating routine prophylaxis, this strategy emphasizes individualized assessment, focusing on patients in whom the potential clinical impact of transient bacteremia may be greater, while avoiding unnecessary antibiotic exposure in low-risk individuals.

Based on these considerations, we propose a multidisciplinary institutional protocol intended to support and structure clinical decision-making, rather than to define mandatory indications. This framework aims to minimize unnecessary antibiotic exposure in low-risk patients while allowing selective prophylaxis in predefined high-risk scenarios. Although the proposed protocol has not been prospectively validated, it may contribute to reducing unwarranted variability in clinical practice.

Future prospective studies are needed to better define the true incidence and microbiological characteristics of infectious complications after catheter ablation and to evaluate the safety, effectiveness, and cost–benefit profile of selective prophylactic strategies. In addition to prospective outcome studies, multicenter cross-sectional surveys investigating current antibiotic prophylaxis practices in electrophysiology laboratories may help quantify real-world variability and identify areas of uncertainty requiring standardization. Such initiatives could represent a first step toward the development of shared, evidence-informed recommendations. Furthermore, the development of validated risk stratification tools integrating patient-related and procedural variables may help refine individualized decision-making; however, any proposed scoring system should undergo rigorous prospective validation before being incorporated into routine clinical practice. Until more robust data become available, pragmatic, risk-based approaches may help guide clinical practice in an area characterized by persistent uncertainty while promoting antimicrobial stewardship.

## 9. Conclusions

In the absence of specific guideline recommendations, routine antibiotic prophylaxis for femoral-access catheter ablation cannot be supported by current evidence. A selective, risk-based approach may represent a reasonable balance between patient safety and antimicrobial stewardship. The multidisciplinary protocol proposed herein offers a pragmatic, adaptable framework intended to support clinical decision-making rather than define universal indications.

## Figures and Tables

**Table 1:** High-risk criteria for selective antibiotic prophylaxis in femoral-access catheter ablation and electrophysiological studies.

High-Risk Domain	Criteria
Intracardiac or intravascular material	Pacemakers, implantable cardioverter-defibrillators, cardiac resynchronization therapy devices, subcutaneous ICDs, cardiac contractility modulation devices, leadless pacemakers, indwelling intracardiac or intravascular leads/catheters, left atrial appendage occlusion devices, patent foramen ovale closure devices
Prosthetic or severe native valvular disease	Mechanical or bioprosthetic valves, annuloplasty rings, severe native valvular heart disease (mitral or aortic) not yet treated, particularly in the presence of additional risk factors.
Immunocompromised status	Solid organ or bone marrow transplantation, onco-hematologic diseases, immunosuppressive therapies, HIV infection with CD4 < 200 cells/mm^3^, chronic dialysis
Previous infection	Documented history of infective endocarditis
Local factors increasing femoral contamination risk	Severe obesity, inguinal skin lesions, urinary or fecal incontinence, compromised hygiene

## Data Availability

Data sharing is not applicable to this article as no new data were created or analyzed in this study.

## Abbreviations

ESC	European Society of Cardiology
EHRA	European Heart Rhythm Association
HRS	Heart Rhythm Society
APHRS	Asia Pacific Heart Rhythm Society
LAHRS	Latin American Heart Rhythm Society
EP	Electrophysiology
CIED	Cardiac Implantable Electronic Device
ICD	Implantable Cardioverter-Defibrillator
HIV	Human Immunodeficiency Virus
CD4	Cluster of Differentiation 4
